# The autophagy elongation complex (ATG5-12/16L1) positively regulates HCV replication and is required for wild-type membranous web formation

**DOI:** 10.1038/srep40351

**Published:** 2017-01-09

**Authors:** Ahmed M. Fahmy, Patrick Labonté

**Affiliations:** 1 INRS-Institut Armand-Frappier, Institut National de la Recherche Scientifique, Laval, Canada

## Abstract

Hepatitis C virus (HCV) infection induces intracellular membrane rearrangements, thus forming a membranous web (MW) in which HCV replication and assembly occur. The HCV-induced MW is primarily composed of double membrane vesicles (DMVs) transfused by multi-membrane vesicles. The autophagy machinery has been proposed to participate in the formation of such vesicles. However, no clear evidence has been found linking autophagy to the formation of these DMVs. In this study, we evaluated the role of the autophagy elongation complex (ATG5-12/16L1) in HCV replication and MW formation. Using a dominant negative form of ATG12 and an siRNA approach, we demonstrated that the ATG5-12 conjugate, but not LC3-II formation, is crucial for efficient viral replication. Furthermore, purification of HCV MW revealed the presence of ATG5-12 and ATG16L1 along with HCV nonstructural proteins. Interestingly, LC3 was not recruited along with the elongation complex to the site of viral replication. Finally, inhibition of the elongation complex, but not LC3, greatly impaired the formation of the wild-type MW phenotype. To our knowledge, this study provides the first evidence of the involvement of autophagy proteins in the formation of wild-type MWs.

Hepatitis C virus (HCV) infection is a leading cause of liver diseases, including cirrhosis and hepatocellular carcinoma. HCV, a member of the *Flaviviridae* family, is a *Hepacivirus* with a positive-strand RNA genome^1^. The virus replicates exclusively in the cytoplasm of the host cell. After cell entry, the 9.6 kb HCV genome is released and translated at the rough endoplasmic reticulum (rER) into a single polyprotein. This translated polyprotein is then proteolytically processed by cellular and viral proteases into 10 distinct proteins consisting of structural (core, E1, and E2) and nonstructural (p7, NS2, NS3, NS4A, NS4B, NS5A, and NS5B) proteins^2^. The expression of HCV proteins results in the induction of a major rearrangement of host cell membranes, thus leading to the formation of a complex membranous compartment termed the membranous web (MW), which favors viral RNA replication and assembly^3^,^4^. This massive remodeling of the host cell membrane network is associated with all positive-strand RNA viruses and is typically characterized by the generation of either convoluted membranes or double membrane vesicles (DMVs)^5^,^6^,^7^,^8^. Importantly, the HCV-induced MW is primarily composed of DMVs thus suggesting that autophagy plays a role in the construction of the HCV replication scaffold^7^,^9^.

Macroautophagy, referred to hereafter as autophagy, is a catabolic pathway that degrades proteins and organelles, thereby maintaining cell homeostasis and directing cell fate. During cellular stress such as amino acid starvation, autophagy is triggered, thereby forming an organelle called the autophagosome. The *de novo* formation of the autophagosome begins by initiation of the growth of a double-membraned phagophore that, by closing, sequesters cytoplasmic contents. The autophagosome then fuses with the lysosome, thus allowing the degradation of the intra-autophagosomal cargo by the action of lysosomal enzymes and the release of free amino acids and other products. This process is orchestrated by more than 30 autophagy-related gene (ATG) proteins and other autophagy-linked proteins^10^.

During the early steps of autophagosome biogenesis, ATG5, ATG12, and ATG16L1 form a stoichiometric complex known as the autophagy elongation complex (ATG5-12/16L1). The elongation complex has been shown to determine the site of LC3 lipidation^11^, a process required for the association of LC3 with the autophagosomal membrane. The membrane-associated LC3 allows the completion of autophagosome formation^12^.

Although autophagy can act as an anti-viral mechanism, many reports have shown that positive-strand RNA viruses, including HCV, can hijack the autophagy machinery for virion morphogenesis and viral replication^13^,^14^,^15^,^16^,^17^. Several studies have shown that HCV induces autophagosome formation *in vitro*^9^,^18^,^19^,^20^,^21^. This finding has also been confirmed in liver biopsies obtained from patients chronically infected with HCV^22^. In addition, HCV infection has been shown to induce mitophagy, the selective removal of mitochondria, through the induction of phosphatase and tensin homolog (PTEN)-induced putative kinase 1 (PINK1) and Parkin on the outer surface of the mitochondria. It has been proposed that degradation of mitochondria enhances HCV replication and suppresses cellular apoptosis^23^. Although several studies have shown that HCV infection induces autophagy by ER-stress via induction of the unfolded protein response (UPR)^20^,^24^,^25^,^26^,^27^, other studies have shown that HCV induces autophagy in a manner independent of ER-stress, through the direct interaction of viral proteins with autophagy proteins^28^,^29^. Moreover, HCV-induced autophagy has been found to play a crucial role in evading host microbial defense mechanisms at the level of innate and adaptive immune responses^30^. The induction of autophagy promotes HCV replication either by enhancing protein translation^31^ or via viral maturation^32^. Owing to the morphological similarities between HCV-induced DMVs and the double-membrane nature of autophagosomes, it has been proposed that autophagy plays a role in the biogenesis of viral replication compartments^7^. Importantly, Atg5 induces the formation of DMVs in embryonic stem cells^33^. In addition, a previous study conducted by our lab has shown that ATG5 interacts with the viral polymerase (NS5B) and co-localizes with NS4B, a MW-associated viral protein^34^. Another group has shown that ATG12, Beclin 1, and ATG4B are required for the establishment of viral replication^31^. However, studies on the involvement of autophagy proteins in the assembly of the MW remain elusive. In this study, we investigated the putative role of the ATG5-12/16L1 complex in the HCV replication-cycle. We found that the autophagy elongation complex (ATG5-12/16L1) is recruited at the MW, where it promotes HCV replication in an LC3-independent manner. Surprisingly, knock-down of one component of the elongation complex, ATG12, led to an aberrant MW phenotype, thus suggesting a novel role of autophagy proteins in the formation of the MW.

## Results

### HCV does not perturb formation of the ATG5-12/16L1 complex

**Figure 1:**
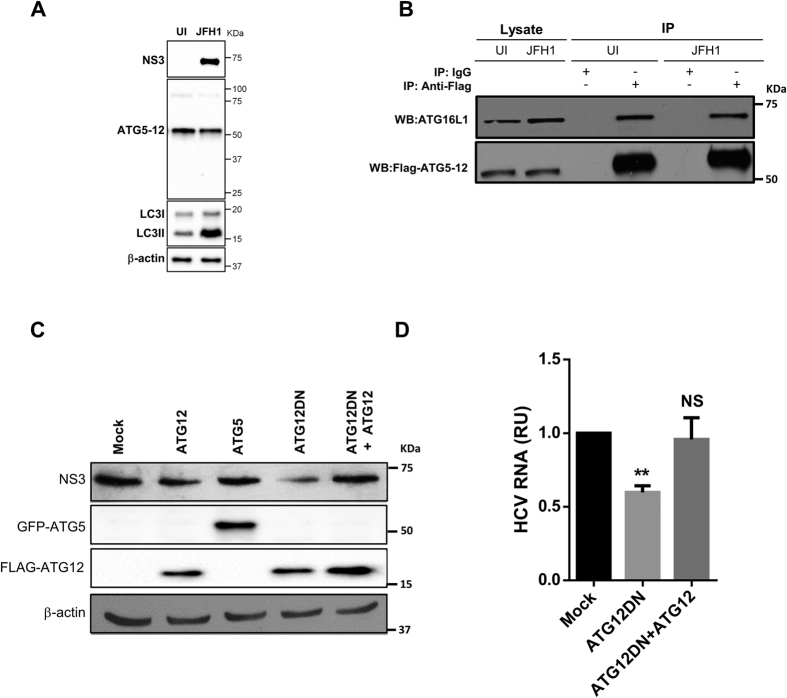
HCV does not alter the formation of the autophagy elongation complex in Huh7 cell. (**A**) ATG5-12 conjugation was assessed by western blotting in mock (UI) or Huh7 cells infected at more than 90% with JFH1 using specific anti-ATG5 antibody. HCV infection and LC3 lipidation were detected using anti-NS3 and anti-LC3 antibodies, respectively. β-actin represents loading control. (**B**) Uninfected or JFH1-infected Huh7 cells at more than 90% were transiently transfected with a plasmid encoding for Flag-ATG12 protein. Two days later, cells were lysed and ATG12 was immunoprecipitated using anti-Flag antibody or IgG as a control followed by western blot analysis using anti-Flag and anti-ATG16L1. (**C**) Huh7 cells were infected with JFH1 (>90% infected) before being transfected with control (mock), Flag-ATG12, GFP-ATG5, Flag-ATG12DN or Flag-ATG12 and Flag-ATG12DN encoding plasmids. Cell lysates were analyzed for HCV NS3, ATG5 and ATG12 protein expressions at 72 h post-transfection by western blotting. (**D**) JFH1-infected cells at more than 90% were transfected with control plasmid (mock), Flag-ATG12DN or Flag-ATG12 and Flag-ATG12DN. Two days later, intracellular HCV RNA was quantified by RT-qPCR. Data were collected from three independent experiments (n = 3). (**P = 0.005, NS, None-significant. Statistical analysis was performed by using One-way ANOVA).

ATG5 forms a conjugate with ATG12, but the monomeric forms of these two proteins have been shown to be nearly undetectable under normal conditions^35^. We first tested whether this conjugation occurs in HCV-infected cells. The assessment of the ATG5 protein by western blotting showed that monomeric ATG5 (32 kDa) was undetectable in both infected and uninfected cells. ATG5 was detected only in the ATG5-12-conjugated form (55 kDa) (Fig. 1A). Infection with HCV JFH1 strain was confirmed by detecting HCV NS3 protein using anti-NS3 antibody (Fig. 1A). The difficulty in detecting unconjugated ATG5 suggested that the majority of the ATG5 is readily conjugated to ATG12 in Huh7 cells, as has previously been reported for other cell types^36^. In addition, HCV infection did not inhibit this conjugation. Furthermore, HCV infection induced LC3-II accumulation (Fig. 1A). This result confirms the capability of HCV to modulate autophagy, as has previously been reported by several groups^9^,^18^,^19^.

After ATG5 is conjugated to ATG12, it forms a multimeric complex by associating with ATG16L1. To test whether this complex forms in HCV-infected cells, we overexpressed ATG12-Flag in infected and uninfected cells. Using co-immunoprecipitation with an anti-flag monoclonal antibody, we detected the ATG5-12/16L1 complex under both conditions (Fig. 1B). Whereas ATG5-12 associates spontaneously with ATG16L1^37^,^38^, our results indicated that HCV infection did not disturb the formation of the ATG5-12/16L1 complex.

### Conjugation of ATG5-12 is required for HCV replication in infected cells

To test whether the decoupling of the ATG5-12 conjugate influences HCV replication, we overexpressed the dominant-negative form of ATG12 (ATG12DN) in HCV-infected Huh7 cells. This mutated form of the ATG12 protein lacks the C-terminal glycine, which is crucial for conjugation with ATG5^39^. Interestingly, the overexpression of this conjugation-defective mutant had an adverse effect on HCV lifecycle, as indicated by a decrease in the NS3 protein (Fig. 1C), as well as the viral RNA level (Fig. 1D), as compared with the levels in mock-treated cells. The specificity of the effect of ATG12DN overexpression was assessed by trans-complementation with wild-type ATG12. As expected, trans-complementation with ATG12 restored the normal level of replication (Fig. 1C,D). Altogether, these results suggest that the ATG5-12 conjugate, rather than the monomeric form of the ATG5 and ATG12 proteins, acts as an HCV proviral factor.

### The ATG5-12 conjugate is involved in the HCV lifecycle at a post-translational step

**Figure 2:**
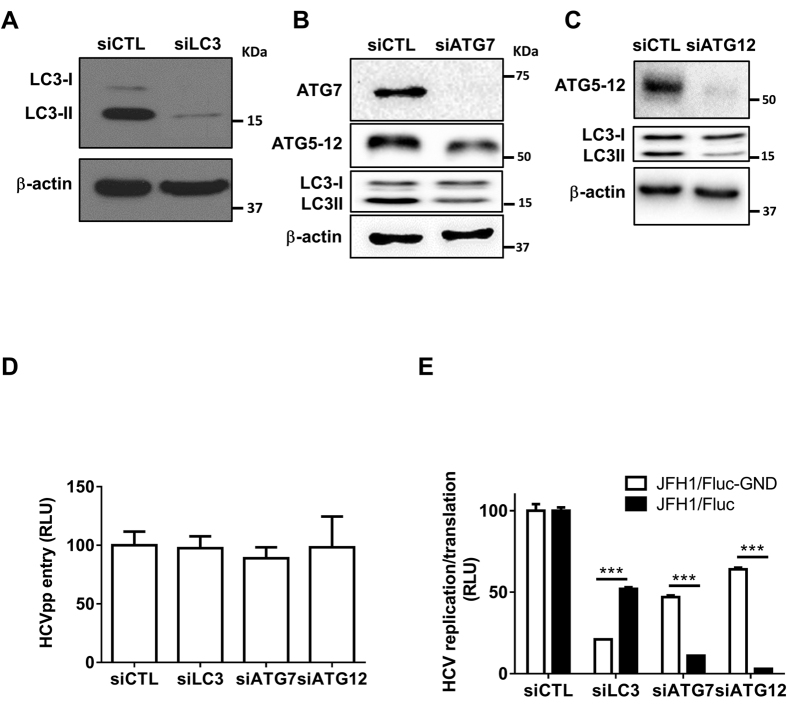
Silencing of ATG7 or ATG12 inhibits HCV lifecycle. (**A**–**C**) Huh7 cells were transfected with siRNA against a non-specific target (siCTL), LC3 (siLC3), ATG7 (siATG7) or ATG12 (siATG12) siRNA, respectively. The cells were lysed 48 h post-transfection and extracts from each treatment were analyzed by western blotting for the corresponding proteins as indicated in the figure. β-actin was used as loading control. (**D**) Huh7.5 cells were transfected with siCTL, siLC3, siATG7 or siATG12. The cells were infected with HCVpp 48 h later and the luciferase activity was assessed 24 h post-infection. Data were derived from two independent experiments (n = 3). (**E**) Huh7.5 cells were transfected with siCTL, siLC3, siATG7 or siATG12 for 48 h and then electroporated with 5 μg of *in vitro*-transcribed viral RNA (JFH1/Fluc or JFH1/Fluc-GND). The cells were lysed 24 h later and the luciferase activity was determined from three independent experiments (n = 3). (***P = 0.001, Statistical analysis was performed by using Student’s-t-test). RLU represents relative luciferase units.

The conjugation of ATG12 to lysine 130 of ATG5 is mediated by ATG7, an E1-like enzyme, thus allowing the formation of the ATG5-12/16L1 complex. A fraction of the ATG5-12/16L1 complex localizes to the isolation membrane, where it facilitates LC3 lipidation. Another role of ATG7, along with ATG3, an E2-like enzyme, is to activate LC3 after its processing at the C-terminus by ATG4B, thus allowing its conjugation to the amino group of phosphatidylethanolamine (PE) and formation of the membrane-associated LC3-II, which assists in the expansion and closure of the autophagosome^40^,^41^. Therefore, silencing of ATG7 allows the inhibition of LC3-II and ATG5-12 conjugation (Fig. 2B). Thus, by silencing LC3, ATG7 or ATG12, we were able to analyze the independent contributions of the two autophagy conjugation systems in the HCV lifecycle. For this purpose, we first determined the efficiency of the selected siRNA to knock down their respective targets (Fig. 2A,B,C). We then analyzed the effects of the silencing of ATG12, LC3 or ATG7 on HCV entry, viral RNA translation/replication and virion maturation and secretion. As shown in Fig. 2D, Huh7 infection by HCVpp was not altered in cells treated with siRNA against LC3, ATG7 or ATG12, thus suggesting that neither LC3 nor the ATG5-12 conjugate is involved in viral entry.

Previously, Dreux and colleagues have reported that HCV RNA translation is affected by inhibition of LC3 conjugation using siRNA against ATG4B. In that report, the authors followed the luciferase activity expressed from a replication-defective subgenomic replicon RLuc/SGR harboring an inactivation mutation (GDD to GND) at the active site of the HCV polymerase NS5B (RLuc/SGR-GND)^31^. Using a similar approach, we evaluated the effects of LC3, ATG7 and ATG12 silencing on viral RNA translation and/or replication. We used full-length HCV JFH1 RNA with a firefly luciferase reporter containing the GND mutation in the active site of NS5B (JFH1/Fluc-GND) and then analyzed the Fluc activity expressed from the HCV internal ribosomal entry site (IRES). A significant decrease in luciferase activity (>80%) was observed in JFH1/Fluc-GND-transfected cells pretreated with siLC3 (Fig. 2E). Silencing of ATG7, which inhibits LC3-II formation as well as ATG5-12 conjugation, decreased viral translation by 50%. This effect probably occurred through the inhibition of LC3-II conjugation, because silencing of ATG12 expression was much less efficient than LC3 silencing at inhibiting viral RNA translation (Fig. 2E). In contrast, silencing of ATG12 severely affected the luciferase activity of a replication competent JFH1/Fluc wild-type virus. The effect on replication was not due to the toxicity of siRNA treatment (Fig. S1), thus suggesting that the ATG5-12 conjugate is involved in an HCV lifecycle step(s) beyond entry and RNA translation (Fig. 2E), whereas LC3 expression and/or conjugation is primarily important for viral translation, as previously suggested^31^.

### The ATG5-12 conjugate positively regulates HCV RNA replication in an LC3-independent manner

**Figure 3:**
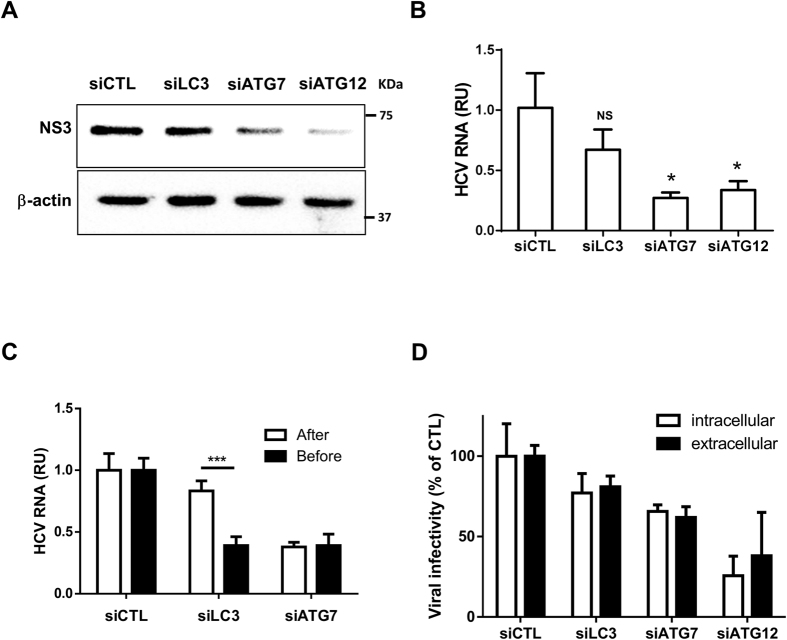
Silencing of ATG7 or ATG12 but not LC3 inhibits HCV replication. (**A**) Huh7 cells stably expressing wild-type JFH1 SGR were transfected with siCTL, siLC3, siATG7 or siATG12 each for 48 h and then cell lysates were analyzed for NS3 by western blotting using specific anti-NS3 antibody. β-actin served as loading control. (**B**) Huh7 cells stably expressing wild-type JFH1 SGR were transfected as in (**A**) and analyzed for SGR RNA by using RT-qPCR. Data are derived from three independent experiments (n = 2). (*P = 0.05, NS, none-significant. Statistical analysis was performed by using One-way ANOVA). (**C**) Huh7 cells were transfected with siCTL, siLC3 or siATG7 prior to or 7 days post-infection with JFH1 (MOI = 0.01). Transfected cells were cultured for 72 h, lysed and subjected to RT-qPCR for intracellular viral RNA quantification. Data are derived from three independent experiments (n = 3) (***P = 0.001, Student’s-t-test). RU represents relative units. (**D**) JFH1-infected Huh7 cells were transfected with siCTL, siLC3, siATG7 or siATG12. Extracellular and intracellular infectivity were determined 48 h post-transfection and expressed as percentage of control. Data shown are from three independent experiments (n = 2).

To determine whether LC3 or the ATG5-12 conjugate modulates HCV RNA replication, we analyzed the effects of silencing these autophagy genes on viral RNA replication in Huh7 cells stably expressing the JFH1 subgenomic replicon (SGR). Using these specific cells, which are capable of only intracellular HCV RNA replication and lacked the capacity to produce infectious viral particles, allowed us to study HCV RNA replication independently of viral entry and egress. Again, silencing of ATG12 but not LC3 efficiently inhibited RNA replication, thus supporting the role of the ATG5-12 conjugate in viral replication and ruling out the possibility that LC3 participates in viral replication (Fig. 3A,B). Because silencing of LC3 expression led to a clear inhibition of HCV RNA translation after electroporation of the viral RNA but did not significantly affect replication in JFH1-SGR cells, we sought to compare the effect of siRNA treatment before and after infection with HCVcc JFH1 (Fig. 3C). The results clearly demonstrated that siLC3 inhibited HCV only when it was transfected before infection, whereas siATG7 was effective when it was transfected before or after infection. These results suggest that LC3 is important early in infection and primarily for initial HCV RNA translation, as has previously been reported^31^. Finally, we evaluated the effects of siRNA treatment on intracellular and extracellular HCV infectious particle production in JFH1-infected cells (Fig. 3D). These results suggested that HCV maturation and secretion was not significantly affected by siRNA treatment. Although silencing ATG12 led to a significant decrease in HCV particle formation, this effect was attributed to a severe reduction in viral replication. Collectively, ATG12 silencing and to a lesser extent ATG7 but not LC3, impaired viral replication.

### ATG5-12 and ATG16L1 are associated with purified MW extract

**Figure 4:**
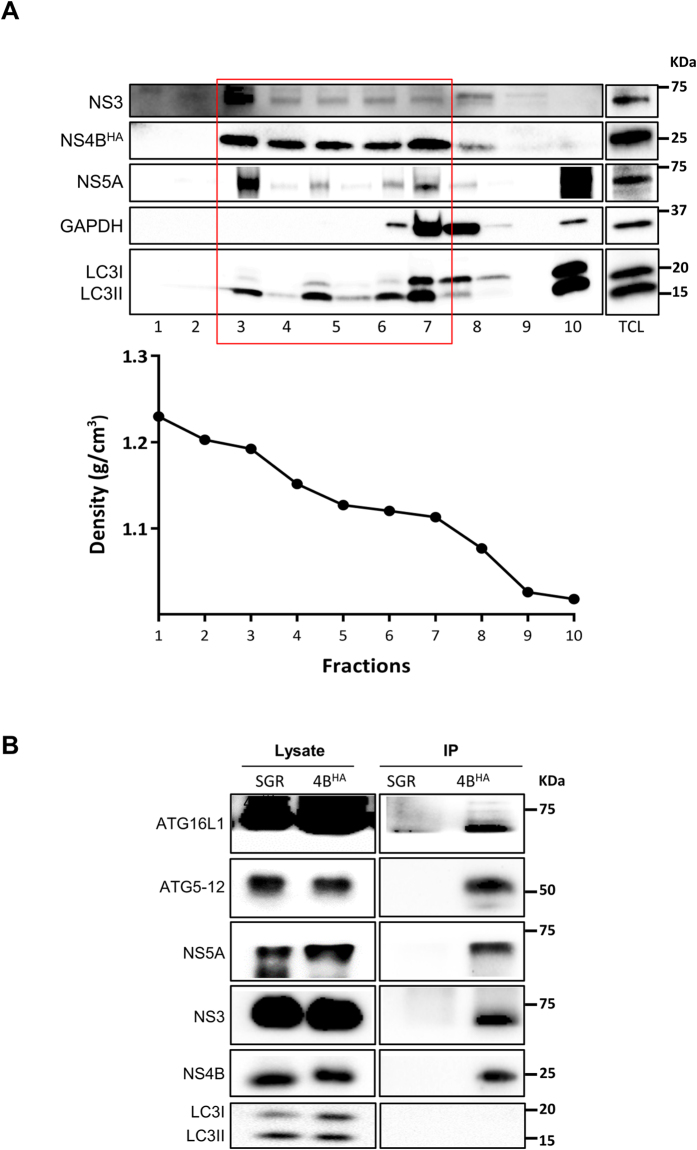
Purified MW extracts harbor the autophagy elongation complex proteins. (**A**) Huh7-Lunet cells harboring HA-tagged NS4B replicons were subjected to sucrose gradient fractionation as described in materials and methods section. The fractions were analyzed for their protein content by western blotting. Specific antibodies were used to detect NS3, NS4B^HA^, NS5A, LC3 and GAPDH as indicated on the left. Fractions containing membrane-associated proteins (boxed in red) were pooled for affinity capture immunoprecipitation. The density of the different fractions is shown in the lower panel. TCL, total cell lysate. (**B**) HA-specific affinity-captured protein content from pooled fraction in (**A**) was analyzed by western blotting to detect viral NS3, NS4B, NS5A and autophagy elongation complex ATG5-12/16L1 by using specific antibodies. Pooled fractions from SGR cell lysate was used to demonstrate pull-down specificity.

**Figure 5:**
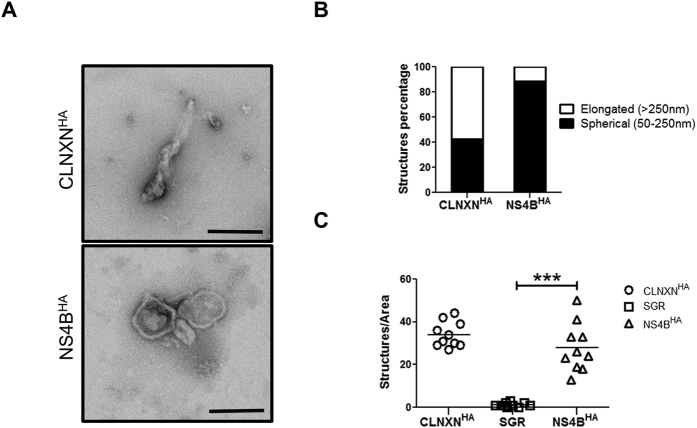
Analysis of the purified MW membranes morphology and number. (**A**) Morphological analysis of HA-captured membranes. HA-captured material from NS4B^HA^ or CLNXN^HA^ were negatively stained and examined using TEM. Scale bars represents 100 nm. (**B**) The purified membranes were categorized into elongated or spherical structures and were represented as relative values. (n > 100). (**C**) Number of membrane structures per area of one hexagon from 10 randomly chosen grid hexagons. (***P = 0.0001, Statistical analysis was performed by using Student’s-t-test).

In a previous study from our lab, we have shown that ATG5 interacts with the HCV polymerase (NS5B) and co-localizes with the MW associated protein NS4B^34^. The major limitation in our ability to investigate the composition of the MW has recently been resolved by Dr. Ralf Bartenschlager’s group, which has developed a method to purify the HCV MW by using HCV replicon cells harboring an HA-tag NS4B (NS4B-HA)^42^. This method allowed us to evaluate the presence of the autophagy elongation complex proteins in purified MW extract. Through this protocol, after membrane enrichment from a discontinuous sucrose gradient via ultracentrifugation, we pooled fractions that were rich in viral nonstructural proteins (fraction 3 to 7) but mostly devoid of soluble proteins (GAPDH or LC3I, fractions 8–10) (Fig. 4A). The MW vesicles were then pulled down from pooled fractions by using a specific antibody against the HA-tag of the HCV NS4B protein. Subsequently, the MW-enriched extract was used for either western blot analysis or vesicle visualization by transmission electron microscopy (TEM). As expected, HCV NS4B^HA^, NS3 and NS5A were readily detectable in the purified extract from NS4B^HA^ replicon cells but not that from control untagged NS4B replicon cells (Fig. 4B). The autophagy elongation complex proteins (ATG5-12 and ATG16L1) were also detected in the purified MW from NS4B^HA^ replicon cells, but not in the control extract, thus indicating that the elongation complex is indeed present at the HCV replication site. In contrast, we were unable to detect LC3II in the purified MW (Fig. 4B), thereby suggesting that LC3 is not recruited with the autophagy elongation complex to the MW. We then examined the morphology of purified membranes and compared them with ER membranes purified from a cell line expressing HA-tagged Calnexin, as previously described^42^. The results showed that almost 90% of the NS4B^HA^ purified membranes were spherical vesicles as compared with CLNXN^HA^ purified material, in which the majority of membranes were composed of partially collapsed large membranes (Fig. 5A,B). Our results are in agreement with those of Paul and colleagues, who have demonstrated that most of the purified ER membranes are composed of elongated structures, as opposed to the spherical vesicles found in MW extracts^42^. Finally, the specificity of the pull-down using HA-beads was confirmed by using extracts from untagged SGR cells (Fig. 5C). Altogether, these results indicate that at least a fraction of the autophagy elongation complex is localized in the virus-induced MW compartments.

### Silencing of ATG12 or ATG7, but not LC3, alters the phenotype of the MW

**Figure 6:**
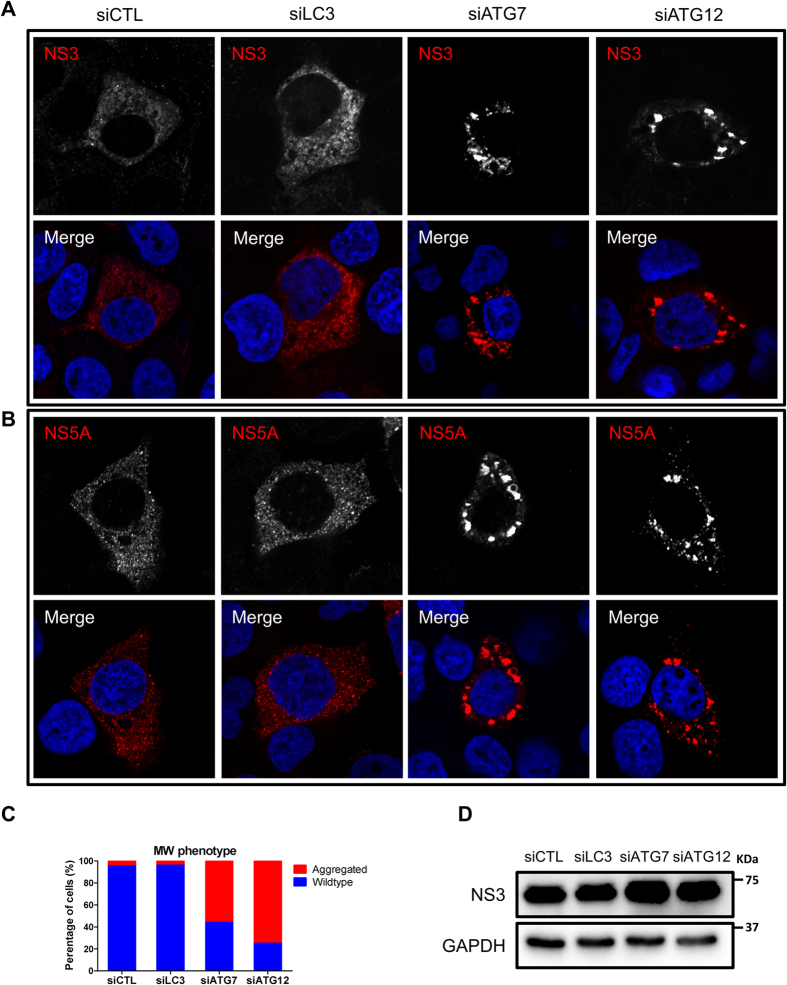
The impact of LC3, ATG7 or ATG12 silencing on membranous web phenotype. (**A**,**B**) Huh7-Lunet-T7 cells were transfected with siCTL, siLC3, siATG7 or siATG12. After 48 h, the cells were transfected with pTM-NS3-5B and were stained 24 h later for NS3 and NS5A using specific antibodies (red). Nuclei were stained with DAPI (blue). (**C**) The percentage of wild-type and clustered phenotype was determined in 100 NS5A-positive cells per condition. (**D**) Huh7-Lunet-T7 cells were transfected with the different siRNAs as in (**A**) and then transfected with pTM-NS3-5B. After 24 h, cells were lysed and examined for HCV NS3 proteins expression by using western blotting. GAPDH was used as loading control.

To evaluate the putative role of host cell proteins in MW formation, Reiss and colleagues have established a T7-polymerase-based HCV RNA synthesis system in which continuous production of HCV polyproteins persists even when HCV replication is abrogated^43^. This system is particularly useful to evaluate the formation of the MW while targeting host cell proteins in the absence of HCV RNA replication. With this system, it has been shown that alterations in MW formation result in a clustered phenotype of HCV nonstructural proteins^43^,^44^. Thus, using the same system (obtained from Dr. Volker Lohmann), we analyzed MW formation indirectly by monitoring viral protein localization after treatment with siLC3, siATG7 and siATG12. Under normal conditions, the NS3 and NS5A cellular distribution appeared as small punctate structures that appeared to be membrane associated (Fig. 6A,B). Treatment with siLC3 did not alter the cellular distribution of the viral proteins, as observed by confocal microscopy (Fig. 6A–C). Strikingly, silencing of ATG7 or ATG12 resulted in the formation of larger protein clusters in most of the transfected cells (Fig. 6A–C). This effect was not due to decreased HCV protein expression level (Fig. 6D), thus suggesting that the ATG5-12 conjugate, but not LC3, is required to obtain a wild-type MW phenotype.

### Silencing of ATG12 or ATG7, but not LC3, modifies MW morphology

**Figure 7:**
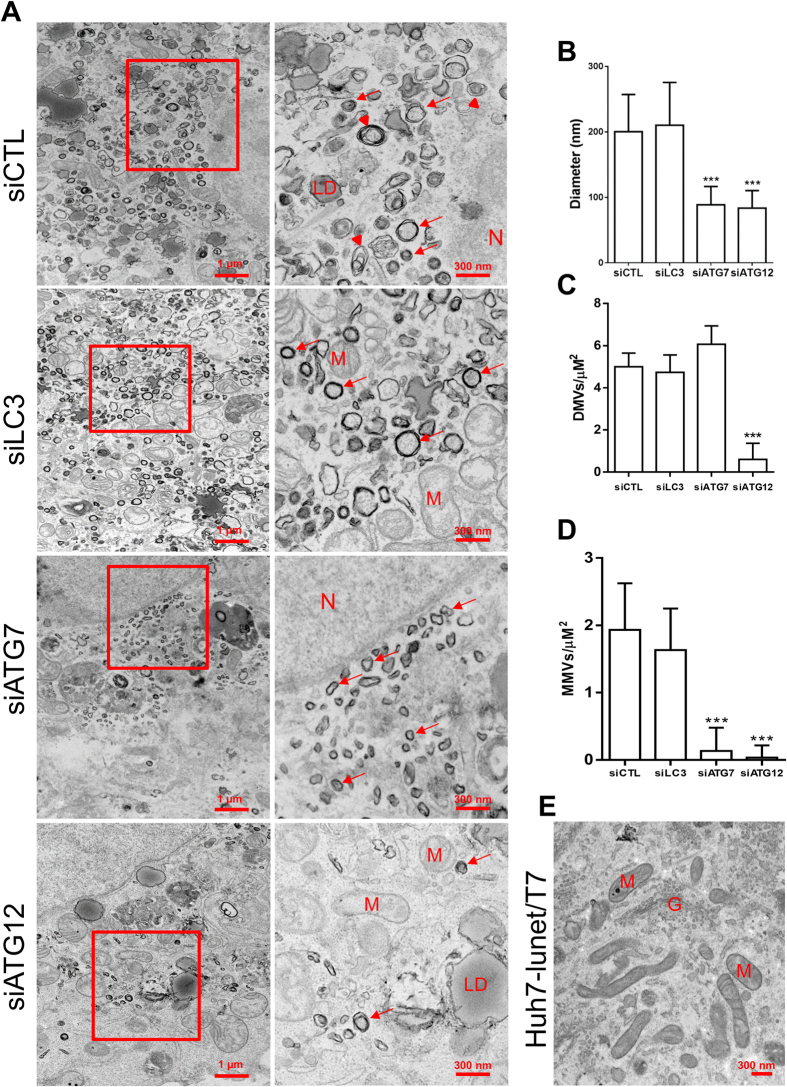
The impact of LC3, ATG7 or ATG12 silencing on membranous web ultrastructure. (**A**) Huh7-Lunet-T7 cells knocked down for LC3, ATG7 or ATG12 were incubated for 48 h prior to transfection with pTM-NS3-5B. After 24 h, cells were fixed and processed for TEM analysis. Lower magnification images and shown on the left with their respective enlargement depicted on the right. Red arrows refer to DMVs. Arrowheads refer to MMVs. N is assigned for nucleus, LD for lipid droplet; M for mitochondria; G for Golgi apparatus. Scale bars are shown in the lower right corner of each panel. (**B**) The average diameter of DMVs assessed in each condition in at least 5 different cells (n = 50 DMVs/cell) (***P = 0.005, Statistical analysis was performed by using One-way ANOVA). (**C**,**D**) The average number of DMVs and MMVs per μM^2^ as calculated from 40 randomly-selected areas per cell (n = 3 cells). (***P = 0.005, Statistical analysis was performed by using One-way ANOVA). (**E**) TEM analysis of naïve Huh7-Lunet-T7 cells.

Next, using TEM, we analyzed MW morphology after treatment with siLC3, siATG7 or siATG12. Expression of pTM-NS3-5B in cells treated with siCTL induced heterogeneous membrane alterations composed of DMVs of an average size of 200 nm interspersed by multi-membrane vesicles (MMVs) that were distributed throughout the cytoplasm (Fig. 7A,B) and that were not seen in negative cells (Fig. 7E). Silencing of ATG7 resulted in more homogenous DMVs with a markedly decreased average size (90 nm) and led to the disappearance of MMVs (Fig. 7A,B,D). Silencing of ATG12 led to a similar effect but with a much lower abundance of DMVs (Fig. 7C). However, silencing of LC3 had no effect on the DMV size (average diameter 200 nm) (Fig. 7B) or on the vesicle types in which both DMVs and MMVs coexist (Fig. 7C,D). Thus, a similar morphology of membrane alterations was observed in siCTL-treated cells (Fig. 7A,B). These results strongly suggest that the ATG5-12 conjugate is crucial for the formation of a typical HCV-induced MW architecture.

## Discussion

In the present study, we demonstrated the requirement of the ATG5-12/16L1 complex for the completion of the HCV lifecycle. HCV infection does not hamper ATG12 conjugation to ATG5 or the formation of the multimeric complex ATG5-12/16L1 (Fig. 1A,B). In contrast, the conjugation of ATG12 to ATG5 is crucial for the HCV lifecycle. More specifically, our study suggests a role of the ATG5-12/16L1 complex in HCV genome replication and the formation of the MW. The involvement of the autophagy elongation complex in the HCV replication step was investigated by using siRNA targeting of ATG7, ATG12 or LC3. Because the silencing of ATG7 is known to inhibit the conjugation of both LC3 and ATG5, we were able to address the importance of these two conjugation systems in HCV replication. Indeed, the ATG5-12 conjugate acted as a proviral factor at a step beyond entry and RNA translation but before virion maturation and secretion, as depicted in Figs 2 and 3. We also observed that silencing of LC3 interfered with HCV RNA translation after electroporation of replication-defective replicon (Fig. 2E). These results are consistent with those of Dreux and colleagues, who have found a defect in viral RNA translation after silencing of Beclin-1 or ATG4B, thus leading to inhibition of LC3-II formation^31^. Silencing of ATG12 had little effect on replication-deficient virus but was detrimental to the replication of the JFH1/Fluc virus, thus indicating that its primary target is beyond the translation step (Fig. 2E). This result was further confirmed in cells stably expressing the JFH1 subgenomic replicon, in which silencing of ATG7 or ATG12, but not LC3, significantly inhibited HCV replication (Fig. 3A,B). Silencing of LC3 impeded HCV only when performed before infection, thus suggesting that the ATG5-12 conjugate, but not LC3, is important in viral replication after the establishment of infection (Fig. 3C).

In addition, the co-purification of the elongation complex proteins with the MW suggested that the ATG5-NS5B interaction previously described by our laboratory^34^ might actually participate in targeting of the elongation complex to the MW and/or in supplying of autophagic isolation membranes for the formation of the virus-induced vesicles. In canonical autophagy, the ATG5-12/16L1 complex is recruited to the isolation membrane prior to LC3 and is released just before the completion of autophagosomes. The absence of LC3 in the purified MW suggests that HCV either hijacks ATG5-12/16L1-positive LC3-negative isolation membranes or initiates the *de novo* formation of the isolation membrane at the MW rather than utilizing LC3-positive autophagosomes for the formation of DMVs within the MW (Fig. 4). Interestingly, the recruitment of the elongation complex to the MW was not accompanied by LC3 lipidation or its relocation at that site. Recently, it has been demonstrated that the ATG5-12/16L1 complex has a membrane-tethering activity that is independent of LC3^45^,^46^. This finding highlights the possibility that in HCV infected cells the major role of the elongation complex is to tether vesicles during MW formation. Concomitantly, it has been reported that some ATG proteins, including ATG16L1, can traffic in LC3-free vesicle-like structures to the site where they probably act to generate *de novo* isolation membranes^47^. This finding also raises the possibility that HCV may recruit similar structures that aid in the formation of the MW.

Recently, Reiss and colleagues have developed a system to evaluate the importance of host factors in membranous web formation^43^. Using this system, we demonstrated that ATG7 as well as ATG12 expression, but not LC3, are important to obtain a wild-type MW phenotype, as observed using confocal microscopy (Fig. 6). Furthermore, the morphology of the HCV-induced vesicles was severely altered after silencing of ATG7 or ATG12, but not LC3. Notably, knocking down ATG12 decreased the size and the number of DMVs, whereas silencing of ATG7 mainly affected their size (Fig. 7). At the moment, it remains unknown whether the altered MW is HCV-replication competent. However, the importance of the ATG5-12 conjugate in HCV RNA replication suggests that the autophagy elongation complex inhibits HCV replication through destabilization of the viral replication factories present within the MW.

In summary, recruitment of the autophagy elongation complex to the MW, which is normally involved in DMV formation, promotes viral replication and maintains proper formation of the wild type MW.

## Methods

### Cell culture and reagents

Huh7 and Huh7-Lunet cells stably expressing Calnexin or NS4B-HA replicon were obtained from Dr Ralf Bartenschlager. Huh7-Lunet cells stably expressing the T7 polymerase (Huh7-Lunet-T7) was obtained from Dr. Volker Lohmann, and the Huh7.5 cell line was obtained from Dr. Charles Rice. All Huh7-derived cell lines were cultured in Dulbecco’s modified Eagle’s medium (DMEM; Gibco) supplemented with 10% v/v fetal bovine serum (FBS) (Multicell), 100 U/ml penicillin, 100 μg/ml streptomycin, and 2 mM L-glutamine (Gibco) at 37 °C, 5% CO_2_, in a humidified incubator. Cell lines harboring the wild-type replicon or NS4B^HA^ were maintained in medium supplemented with G418 (Gibco) at a final concentration of 500 μg/ml. Huh7-Lunet-T7 and Huh7-Lunet cells stably expressing calnexin were cultured in the presence of 10 μg/ml blasticidin (*In vivo* Gen).

### Plasmids and antibodies

The hATG5 and hATG16L1 sequences were cloned into the peGFP-C1 plasmid (Clontech), thus forming pGFP-ATG5 and pGFP-ATG16L1, respectively. The Flag-tagged ATG12 (pATG12) and its dominant-negative derivative pATG12ΔG140 (ATG12DN) constructs were kindly provided by Dr. Adi Kimchi^39^. The PTM vector for the expression of HCV nonstructural proteins NS3 to 5B (pTM-NS3-5B) was kindly provided by Dr. Volker Lohmann. Rabbit polyclonal anti-LC3, rabbit polyclonal anti-ATG5, mouse monoclonal anti-Flag, and mouse monoclonal anti-β-actin antibodies were purchased from Sigma Aldrich. Rabbit polyclonal anti-ATG12 and anti-ATG7 were purchased from Cell Signaling. Rabbit polyclonal anti-ATG16L1 antibody was purchased from MBL. Mouse monoclonal anti-HA was purchased from Roche. Mouse monoclonal anti-NS3 and anti-NS5A antibodies were purchased from BioFront. Rabbit polyclonal anti-NS3 and NS5A were obtained from Dr. Olivier Nicolas. Rabbit polyclonal anti-NS4B and anti-NS5B antibodies were kindly provided by Drs. Kouacou Konan and Takaji Wakita, respectively. Mouse monoclonal anti-GAPDH was purchased from Santa Cruz.

### Preparation of viral stock and infections

The cell culture-derived HCV (HCVcc) JFH1 virus was generated in Huh7 cells by transfection of *in vitro-*transcribed full-length JFH1 RNA (MEGAscript, Ambion). Viral stocks were produced by infection of Huh7 cells at a multiplicity of infection (MOI) of 0.01, as described previously^48^. A replicative bicistronic JFH1-based full-genome construct expressing Firefly luciferase (pJFH1/Fluc) and a clone with a mutation in the viral polymerase (GDD-to-GND) (pJFH1/Fluc-GND) were generated as previously described^49^. To reach 90% infected cells, Huh7 cells were infected at an MOI of 0.01, passaged for 7 days and then analyzed by immunofluorescence using an anti-NS5A antibody.

### Western blot analysis

Cells were lysed in 300 μl of lysis buffer [25 mM Tris-HCl, 150 mM NaCl, 1 mM EDTA, 1% NP40, Complete protease inhibitor (Roche)]. The lysates were normalized for total protein content using the BCA protein assay kit (Pierce). The proteins were then resolved by SDS-PAGE, transferred to polyvinylidene fluoride (PVDF) membranes (Bio-Rad), blocked for 30 min at room temperature (RT) with PBS-5% milk, and then incubated overnight at 4 °C with primary antibody in PBS-2% BSA. After being washed with 0.4% Tween 20 in PBS (PBST), the membranes were incubated for 1 h at RT with a goat-anti-rabbit or goat-anti-mouse IgG conjugated to horseradish peroxidase in PBS-5% milk. Protein bands were visualized with either the Clarity western ECL (Bio-Rad) or Femto chemiluminescence substrates (Pierce).

### Immunoprecipitation

HCVcc-infected and uninfected Huh7 cells were transfected with a plasmid encoding ATG12-Flag. At day 2 post-transfection, the cells were placed on ice and washed with PBS containing 1 mM Na_3_VO_4._ The cells were scraped in the presence of 300 μl of lysis buffer [1% NP-40, 20 mM Tris-HCl (pH 7.5), 135 mM NaCl supplemented with 50 mM NaF, 10 mM Na_4_P_2_O_7_, 1 mM Na_3_VO_4_, 1.5 mM EGTA and Complete™ protease inhibitor (Roche)]. ATG12-Flag was immunoprecipitated from the total lysate by using anti-Flag antibody.

### Purification of HCV-induced MW

HCV-remodeled membrane purification was performed using a method adopted from a previously described protocol^42^. Briefly, 7.5 × 10^7^ Huh7-Lunet cells harboring either wild-type or HA-tagged NS4B replicons or control cells stably overexpressing CANX^HA^ were washed, scraped and then resuspended in 500 μl of hypotonic buffer and incubated on ice for 30 min. The cells were lysed with 50 strokes with a Dounce homogenizer. The lysates were centrifuged at 800×g for 10 min at 4 °C. Supernatants were collected and layered on top of a discontinuous sucrose gradient (70% to 30%) and centrifuged at 130,000×g for 4 h at 4 °C using an SW60i rotor (Beckman Coulter). Ten fractions were collected from the bottom (300 μl each) and analyzed for protein content. For HA affinity capture, fractions 3 to 7 were pooled, and then an equal amount of protein contained in pooled fractions was equilibrated to 150 mM NaCl. Incubation with HA-agarose beads (Sigma-Aldrich) was performed as previously described^42^.

### Membrane visualization by transmission electron microscopy

To examine purified membranes, 50 μl of eluted material was centrifuged at 10 p.s.i. on a copper grid for 5 min at RT in an Airfuge (Beckman). Structures were negatively stained using 2% aqueous uranyl acetate for 30 sec and examined with an H-7100 (Hitachi) transmission electron microscope.

### Quantification of HCV RNA by RT-qPCR

Isolated RNA was reverse transcribed with M-MLV (Invitrogen). The generated cDNA was used for qPCR using Taqman probes, as previously described^50^. Results were analyzed using the comparative ΔCt method.

### Small interfering RNA (siRNA) transfection

Huh7 cells were reverse transfected in a 24-well plate with siRNA (25 nM final concentration) using Lipofectamine RNAiMAX reagent (Life Technologies) according to the manufacturer’s protocol. Huh7 cells were transfected with siRNA to GFP, LC3B siRNA (UACCUGUAUACGUUAGUGAAAUU) or with an ON-TARGETplus human ATG7 siRNA-SMART pool (catalog no. L-020112-00-0005). To study the onset of replication, Huh7.5 cells were reverse transfected with siRNA, as described above, in 6-well plates. Forty-eight hours later, the cells were trypsinized, washed twice with cold PBS, resuspended in 100 μl of cold Ingenio electroporation solution (Mirus) and then electroporated with 5 μg of *in vitro*-transcribed viral RNA (JFH1/Fluc or JFH1/Fluc-GND) in 2 mm gap electroporation cuvettes by using a BTX Harvard Apparatus with the following settings: 820 V, 99 μS, 4 pulses, 1.1 s interval. The cells were then resuspended in DMEM-10% FBS and seeded in 96-well plates and further cultured for 24 h. The cells were then lysed in 20 μl luciferase lysis buffer (RLB) and stored at −80 °C until measurement of luciferase activity. For the determination of intra- and extracellular virus titers, JFH1-infected Huh7 cells were reverse transfected with siRNA in 6-well plates. Two days later, the cells were washed three times with PBS and supplemented with fresh DMEM. After 24 h, cells and supernatants were harvested. The cells were washed twice with PBS, trypsinized, resuspended in 1 ml culture medium and subjected to 3 rapid freeze-thaw cycles in a dry ice/ethanol bath and 37 °C water bath, respectively. Cell debris was removed by centrifugation at 10,000 rpm for 3 minutes. Samples were analyzed using a limiting dilution assay.

### Production of HCVpp and cell entry assay

Viral pseudotyped particles harboring the HCV glycoproteins (HCVpp) were produced by transfection in Hek-293T cells of vectors encoding viral glycoproteins, packaging proteins and a Luciferase marker. After 48 h, viral pseudoparticle supernatants were harvested and filtered through 45-μM filters to remove the cell debris. For the entry assay, Huh7.5 cells were reverse transfected with siRNA in 96-well plates. After 48 h, the cells were infected with 50 μl HCVpp containing supernatant. Forty-eight hours post-infection, the cells were washed three times with PBS, lysed in 20 μl luciferase lysis buffer (RLB) and stored at −80 °C until measurement of luciferase activity.

### Luciferase assay

Cell lysates were prepared with Reporter Lysis Buffer (RLB) (Promega), and luciferase activity was measured with a luciferase assay system (Promega), per the manufacturer’s protocol.

### Assessment of the MW phenotype

For the immunofluorescence experiment, Huh7-Lunet-T7 cells were reverse transfected with siRNA as previously described^43^. Forty-eight hours later, a second round of transfection with siRNA was performed. After 48 h, cells were transfected with pTM-NS3-5B using Lipofectamine 3000 (Invitrogen). The coverslips were then fixed with 4% formaldehyde in PBS for 10 min, washed in PBS and incubated in blocking buffer (PBS, 3% bovine serum albumin, 10% FBS, 0.1% Triton X-100) for 30 min at RT. After being washed three times with PBS, the coverslips were incubated with primary antibody in blocking buffer for 1 h at RT. Then, the coverslips were washed with PBS and incubated with either Alexa Fluor™-(488 or 568) goat anti-mouse IgG or Alexa Fluor™-(488 or 568) goat anti-rabbit IgG (Invitrogen) for 1 h at RT. After being washed, the coverslips were mounted on glass slides with Prolong™. Antifade (Invitrogen) and examined with a laser scanning confocal Zeiss LSM 780.

For TEM analysis, a similar setup was used, except that after transfection with pTM-NS3-5B, the cells were trypsinized and seeded into lab-tek chamber slides (Thermo Fisher). After 24 h, the monolayer of cells was washed with PBS, fixed with 2.5% glutaraldehyde (Electron Microscopy Science) and incubated overnight at 4 °C. The cells were then washed in 0.1 M cacodylate (Electron Microscopy Science) and incubated in 1% osmium tetroxide (Mecalab) for 1 h at 4 °C. The cells were dehydrated in a graded series of ethanol/deionized water solutions (from 50% to 100%). The cells were then infiltrated with a 1:1 and 3:1 Epon 812 for 1 h for embedding and polymerized overnight in an oven at 60 °C. The polymerized blocks were trimmed, and 100 nm ultrathin sections cut with an UltraCut E ultramicrotome (Reichert Jung) and transferred onto 200-mesh copper grids (Electron Microscopy Science) with formvar support film. The sections were stained with 4% uranyl acetate (Electron Microscopy Science) for 8 min, then with lead citrate for 5 min (Fisher scientific). The cells were imaged with an FEI Tecnai 12 transmission electron microscope (FEI company) operating at an accelerating voltage of 120 kV and equipped with an AMT XR80C CCD camera. Vesicle size was measured using Image J (NIH).

### Cell viability assay

Cells were reverse transfected with different siRNAs used in this study in a 96-well plate for 48 h. Cell viability was then assayed using the CellTiter 96^®^ AQueous Non-Radioactive Cell Proliferation Assay reagent (Promega).

### Statistical analyses

The results shown represent the mean of at least three independent experiments. Student’s-t-test and one-way ANOVA with Dunnett’s post-test (as indicated in the figure legends) were performed using GraphPad Prism 5. P-values below 0.05 were considered statistically significant.

## Additional Information

**How to cite this article**: Fahmy, A. M. and Labonté, P. The autophagy elongation complex (ATG5-12/16L1) positively regulates HCV replication and is required for wild-type membranous web formation. *Sci. Rep.* **7**, 40351; doi: 10.1038/srep40351 (2017).

**Publisher's note:** Springer Nature remains neutral with regard to jurisdictional claims in published maps and institutional affiliations.

## Supplementary Material

Supplementary Figure 1
