# Supplementary material for: The autophagy elongation complex (ATG5-12/16L1) positively regulates HCV replication and is required for wild-type membranous web formation
Source: Sci Rep. 2017 Jan 9;7:40351. doi: 10.1038/srep40351 (PMC5220323; doi:10.1038/srep40351)
Supplement: Supplementary Figure 1 [file srep40351-s1.pdf]

The autophagy elongation complex (ATG5-12/16L1) positively regulates HCV replication and is required for wild-type membranous web formation

Ahmed M Fahmy and Patrick Labonté

Supplementary materials

Supplementary Figure 1

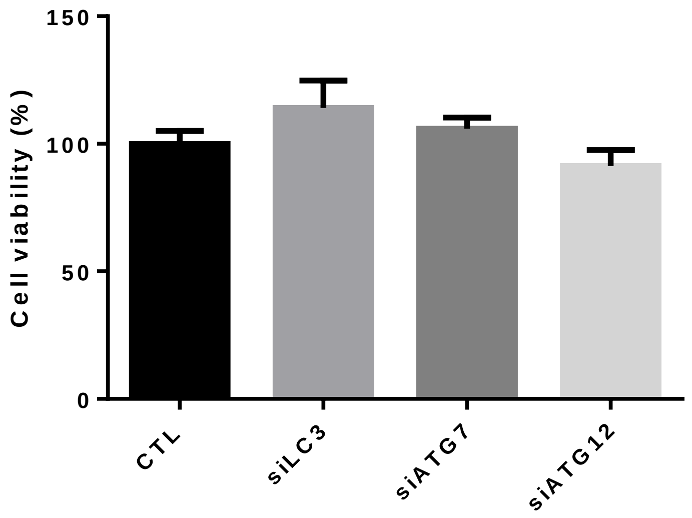

Figure S1. Cell viability of Huh7 cells treated with different siRNAs.

Naïve Huh7 cells were transfected with siCTL, siLC3, siATG7 or siATG12. After 48 h, cells were incubated with CellTiter 96® AQueous reagent for 2 h and the optical density was measured according to the manufacturer's protocol. (n=4).
